# Successful treatment of cerebral aspergillosis: case report of a patient with T-cell large granular lymphocytic leukemia (T-LGL)

**DOI:** 10.1186/s12879-017-2877-8

**Published:** 2017-12-28

**Authors:** Amin T. Turki, Jassin Rashidi-Alavijeh, Jan Dürig, Guido Gerken, Peter-Michael Rath, Oliver Witzke

**Affiliations:** 1 Department of Gastroenterology and Hepatology, University Hospital Essen, Essen, Germany; 2 Department of Bone Marrow Transplantation, West-German Cancer Center, University Hospital Essen, Essen, Germany; 3 Department of Hematology, West-German Cancer Center, University Hospital Essen, Essen, Germany; 4 Institute of Medical Microbiology, University Hospital Essen, Essen, Germany; 5 Department of Infectious Diseases, University Hospital Essen, Essen, Germany

**Keywords:** Invasive aspergillosis, *Aspergillus*, T-LGL, Cerebral abscess, Voriconazole, Amphotericin B

## Abstract

**Background:**

Invasive aspergillosis involving patients with neutropenia or severe immunosuppression, such as patients with hematologic malignancies is associated with high mortality. Patients with T-cell large granular lymphocytic leukemia (T-LGL) on the other hand are considered to be less vulnerable for severe opportunistic fungal infection as their course of disease is chronic and marked by less violent cytopenia then in e.g. Aplastic Anemia. Only neutropenia is regarded as independent risk factor for severe opportunistic infection in T-LGL patients.

**Case presentation:**

We report a case of a 53 year old patient with T-LGL, Immune-Thrombocytopenia (ITP) and combined antibody deficiency, who presented with fever and reduced general condition. The patient revealed a complicated infection involving the lungs and later the brain, with the presentation of vomiting and seizures. Broad microbiological testing of blood-, lung- and cerebrospinal fluid samples was inconclusive. In the absence of mycological proof, *Aspergillus* infection was confirmed by pathological examination of a brain specimen and finally successfully treated with liposomal amphotericin B and voriconazole, adopting a long-term treatment scheme.

**Conclusions:**

Beyond typical problems in the clinical practice involving fungal infections and hematologic malignancies, this case of invasive aspergillosis in a patient with T-LGL illustrates caveats in diagnosis, therapy and follow-up. Our data support careful ambulatory monitoring for patients with T-LGL, even in the absence of neutropenia. Especially those patients with combined hematologic malignancies and immune defects are at risk. Long-term treatment adhesion for 12 months with sufficient drug levels was necessary for sustained clearance from infection.

## Background

In patients with severe immunosuppression or neutropenia, such as patients with hematologic malignancies [1, 2] or HIV [3, 4] invasive aspergillosis is associated with high morbidity and mortality. Infection is regularly acquired via airway inhalation of *Aspergillus spp.* affecting the pulmonary tract, but can disseminate to other organs, such as kidneys or brain [5]. Cerebral aspergillosis is associated with vascular complications [6] and particularly high mortality [7–9]. Combined immunodeficiency syndromes include congenital disorders such as Common Variable Immunodeficiency (CVID) and acquired disorders such as Aplastic Anemia. Patients with T-cell large granular lymphocytic leukemia (T-LGL) on the other hand are considered to be less vulnerable for severe opportunistic fungal infection [10]. T-LGL is a rare hematological condition involving T-cell receptor (TCR) rearrangement and functional T-cell deficiency, often associated with signal transducer and activator of transcription 3 (STAT3) mutation, described in 20–40% of patients with T-LGL [11]. Mutation-induced molecular signaling leading to chronic cell activation and immune-impairment is also observed in other lymphoma, associations between T-LGL and other hematologic disorders have been previously described [12]. While the course of disease in T-LGL is often chronic and marked by less violent cytopenia then in e.g. Aplastic Anemia, the treatment and follow-up can be complicated. Even in the absence of neutropenia, T-LGL patients with combined immunodeficiency or associated hematologic disorders, such as immune-thrombopenia (ITP), may experience complicated infections.

## Case presentation

**Fig. 1:**
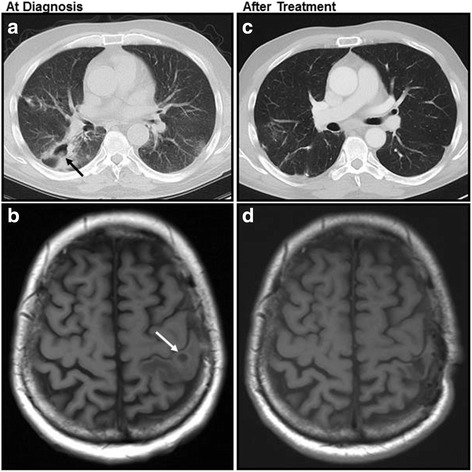
CT-scan of the lungs and MRI of the brain in early stage of disease (**a**, **b**) and after treatment (**c**, **d**), showing bilateral infiltrates and cavitations (**a**, black arrow) and cerebral abscess (**b**, white arrow). After therapy, only residual defects can be observed (**c**, **d**)

A 52-year-old Kosovo man in reduced general condition with fever up to 39.5 °C was admitted to a small urban German hospital. He reported cephalgia and strong, non-productive cough. Since three years, he was known to suffer from ITP, initially treated with corticosteroids (1 mg/kg prednisone) and after relapse with splenectomy six months ago, resulting in sustained remission. A year ago, the patient was further diagnosed with T-LGL having a clonal expansion of CD3/CD5/CD8/CD57 positive T-cells in the peripheral blood. Typical TCR-B und TCR-G rearrangement was detected, but no activating somatic STAT3 mutation. He also had an insulin-dependent diabetes mellitus type 2, hypertonia and drug allergies to penicillins and sulfonamides. On admission, the patient reported he had quit long-term smoking (100 packyears) three years ago. The clinical examination revealed basal crackles on both lungs, concordant with bilateral inflammatory infiltrates in the chest radiography (CXR). The CT-scan (Fig. 1a) confirmed the diagnosis of an atypical pneumonia with possible fungal involvement (according to modified EORTC guidelines: [13]). At admission laboratory testing showed leukocytosis (23.8/nl, normal range: 3.6–9.2/nl, 40% neutrophils), mild anemia (10.1 g/dl, normal range 13.7–17.2 g/dl), elevated CRP (8.6 mg/dl, normal: < 0.5 mg/dl; procalcitonin was negative) and combined antibody deficiency (e.g. serum IgG of 4.0 g/l (normal range: 7.0–16.0 g/l). The abnormal cellular immunogram had a peak of CD8-positive T-cells (7400/μl, normal range: 201–735/μl), in particular CD3/5/8/57+ T-cells with TCR (CD3) rearrangement. Bronchoscopy showed an inflamed right lower lobe with strong fluid formation, putrid secretion and tracheitis. Microbiological analysis of the bronchoalveolar lavage (BAL) detected *Serratia rubidaea*, *Escherichia coli,* and *Aspergillus fumigatus*. Cytological examination revealed lymphocytosis, neutrophilia and discrete eosinophilia. The patient was treated with intravenous imipenem (500 mg 4×/d), ciprofloxacin (400 mg BID) and voriconazole (200 mg BID) for suspected atypical pneumonia. Bacteriological susceptibility testing confirmed antibiotic treatment. Mycological culture and susceptibility testing failed. Standard blood cultures, tuberculosis diagnostics and galactomannan tests were negative. Because of his immunodeficiency, intravenous immunoglobulin (IVIg monthly) was prescribed. Evaluated by CT-scan, the infiltrates alleviated, although residual cavitations remained. With this favorable course of disease, the patient was discharged and instructed to continue oral medication with ciprofloxacin and voriconazole for two more weeks.

**Fig. 2:**
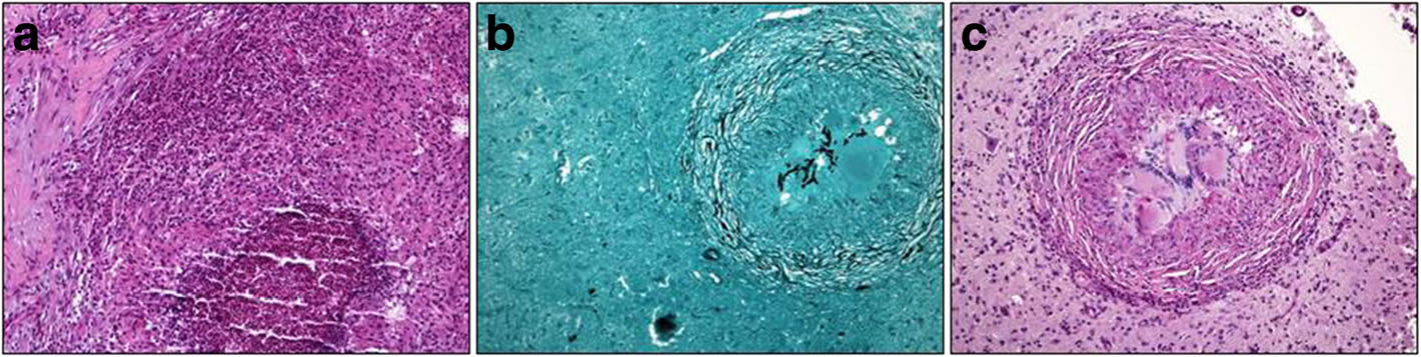
Brain tissue with necrotizing granulomatous inflammation and filamentous fungi, most likely *Aspergillus* spp. **a** HE, **b** Grocott, **c** PAS

About one week after discharge, the patient presented with neurologic symptoms including acute strong nausea, vomiting, fever, relapsing focal seizures of his right arm, paresthesia and motoric weakness. After admission to the same small hospital, he was immediately transferred to our center, the University Hospital of Essen. Upon arrival, his poor clinical status was unchanged. Seizures were successfully treated with levetiracetam. The cerebrospinal fluid (CSF) was normal except for a discrete monolymphocytic reaction. CSF culture, galactomannan- and PCR tests were all negative. MRI of the cranium revealed multiple cerebral abscesses (Fig. 1b). The brain lesions’ etiology being unclear, antibiotic treatment was escalated to linezolid (600 mg BID) and meropenem (1 g 3×/d) to cover a possible multi-resistant nosocomial cerebral infection. The measured voriconazole serum level were insufficient (< 0,5 mg/l; recommended 1–6 mg/l) [14], voriconazole dosage was adjusted (from 200 mg to 400 mg BID) according to current guidelines. The patient was continuously treated with a combination of linezolid, meropenem and voriconazole for about 6 weeks. While his clinical status slightly bettered, the brain MRI and the chest CT-scan did not significantly improve. Therefore, the diagnosis was questioned and a lung sample obtained by mini-thoracotomy in order to identify the pathogen. The microbiological analysis of the sample was negative. Histological analysis supported the diagnosis of chronic organizing pneumonia but remained inconclusive. Hence, a brain tissue sample was obtained through stereotactic puncture of a central lesion in the left hemisphere. The histological analysis revealed a necrotizing granulomatous inflammation with detection of fungal mycelium, most likely *Aspergillus* species (Fig. 2). Proven cerebral aspergillosis along with involvement of the lungs explained the observed symptoms. Because of previously reported inadequate voriconazole serum levels and insufficient treatment response including cerebral progress despite on-going medication, the treatment was switched to liposomal amphotericin B (3 mg/kg, 250 mg/d). Under this therapy, both lesions in the lungs and in the brain regressed and the patient’s clinical status bettered. Because of complications including acute renal failure (stage II), the medication was switched back to voriconazole (at 400 mg oral BID). Frequent voriconazole serum level controls were assured (all in the range of 2 to 4 mg/l). Under this medication the patient’s status further improved. He was discharged and instructed to compliantly stay on therapy. Voriconazole treatment was continued for a total of 12 months. At therapy completion, imagery of brain and lung was freed from *Aspergillus* abscesses or cavitary lesions (Fig. 1c, d). Over a follow-up period of three years the patient presented at the outpatient clinic in a good general condition (ECOG 1), his hematologic conditions remained controlled with a constantly elevated CD8 fraction in the peripheral blood.

## Discussion and conclusions

**Fig. 3:**
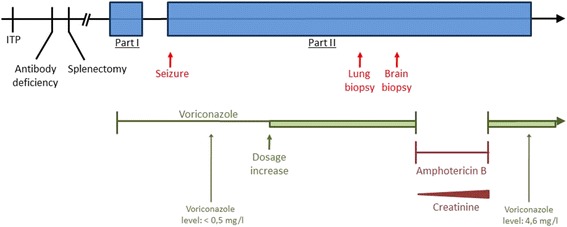
Timeline of disease and treatment periods

Even in the absence of neutropenia, T-LGL patients with combined immune-disorders require intensive infection-monitoring. Beyond typical problems in the clinical practice involving fungal infections and hematologic malignancies, this case of successful treatment of invasive aspergillosis in a patient with T-LGL illustrates caveats in diagnosis, therapy and follow-up. The patient’s initial workup was incomplete at University hospital contact, his aspergillosis manifested sequentially in two different organs, diagnosis was only confirmed histologically and treated with two different antifungal agents (a treatment overview is illustrated in the timeline, Fig. 3). The diagnostic process followed a multi-level approach involving clinical, radiological, microbiological and pathological findings. While the first site of infection was the lung, the disease spread to the brain.

EORTC guidelines distinguish between possible, probable and proven fungal infection as infection can only be proven in <50% of all cases [15]. Uncertainty on our patient’s aspergillosis diagnosis remained until histologic proof was obtained. While low galactomannan levels have been claimed to be favorable predictors [16], our patient’s testing was negative despite his complicated clinical picture. It is well known, that galactomannan detection is more sensitive in BAL than in serum. In a series of patients with hematologic malignancies serum galactomannan was only positive in 43% of patients with other mycological evidence of invasive aspergillosis [17]. The sensitivity of galactomannan detection in CSF is less clear. There are some case series indicating, that a positive result is helpful in the diagnosis of cerebral infection [18]. In inconclusive clinical settings, invasive diagnostic procedures need to be adopted. In our case, two organs were biopsied in order to prove the infection and treat it adequately. The question of early invasive detection in the diagnosis of aspergillosis is open. While in reported cases of CNS processes biopsies associated with better outcome [8], a randomized controlled trial with hematologic patients showed no clinical benefit for early invasive lung diagnosis using BAL [19]. Again, data from other authors supported early invasive diagnostics in lung aspergillosis [20]. As a fraction of T-LGL patients share a combined immunodeficiency involving T cells, bone-marrow suppression and associated disorders, such as in our case ITP and immunoglobulin-deficiency, they represent a collective at risk for severe fungal infections with complicated presentations. While neutropenia is widely accepted as mortality risk factor in T-LGL [10, 21], fungal infection appears underestimated, especially when diagnosis is difficultly established like in our case. An alternating therapy with voriconazole and liposomal amphotericin B was adopted due to complications including inappropriate drug serum levels and acute renal failure. The efficiency of liposomal amphotericin B in CNS aspergillosis is controversial. It poorly penetrates the blood-brain-barrier resulting in low CSF drug concentrations [22]. On the other hand, high concentrations of liposomal Amphotericin B have been measured in post-mortem brain tissue analyses despite low liquor concentrations [23]. Other cases reported successful treatment with voriconazole in immunocompromised patients [24, 25]. Voriconazole effectively penetrates the blood-brain barrier. Randomly-collected CSF samples from patients receiving voriconazole confirmed in most tests drug concentrations exceeding MIC_90_ values for Aspergillus [26]. Previously, the importance of dose escalation in cerebral aspergillosis has been highlighted in order to achieve a satisfying treatment result [27]. First-line treatment with voriconazole is considered superior to amphotericin B and hence recommended in patients with hematologic malignancies [28]. Retrospectively, the switch from voriconazole to liposomal amphotericin B may be questioned but was justified by highly variable voriconazole serum levels and a critical clinical picture despite on-going treatment during the early in-patient phase. Voriconazole has a nonlinear pharmacokinetic profile resulting in extreme interpatient variability [29, 30], especially in patients with liver disease [31], CYP450-mediated drug-drug-interactions [32] and patients of different age [33]. Efficient drug monitoring has been previously reported to be important [34]. During the second voriconazole treatment period elevated serum levels were maintainable. Treatment adhesion and regular measurement of sufficient serum drug levels were crucial to success. The minimal treatment duration is still controversial and varies between weeks and years, depending on the primary site of invasive aspergillosis. In cerebral involvement, prolonged treatment is recommended [27]. During his long-term follow-up of now over three years, our patient showed no further relapse after 12 months of effective voriconazole treatment.

Our case supports careful ambulatory monitoring for patients with indolent lymphoma such as T-LGL. Especially those patients with combined hematologic malignancies and immune defects are at risk for opportunistic infections. Diagnosis of aspergillosis can be challenging and necessitate invasive diagnostics. In our case with invasive cerebral aspergillosis, long-term treatment adhesion for 12 months with sufficient drug levels was necessary for clearance from infection.

## Abbreviations

BID: Twice a day
CNS: Central nervous system
CRP: C-reactive protein
CSF: Cerebrospinal fluid
CT: Computed tomography
CXR: Chest radiography
ECOG: Eastern Cooperative Oncology Group
HE: Hematoxylin and eosin stain
ICU: Intensive care unit
ITP: Immune Thrombocytopenia
MRI: Magnetic Resonance Imaging
PAS: Periodic acid–Schiff
STAT3: Signal transducer and activator of transcription 3
TCR: T-cell receptor
T-LGL: T-cell large granular lymphocytic leukemia
